# Gene Expression Changes Induced by *Trypanosoma cruzi* Shed Microvesicles in Mammalian Host Cells: Relevance of tRNA-Derived Halves

**DOI:** 10.1155/2014/305239

**Published:** 2014-04-09

**Authors:** Maria R. Garcia-Silva, Florencia Cabrera-Cabrera, Roberta Ferreira Cura das Neves, Thaís Souto-Padrón, Wanderley de Souza, Alfonso Cayota

**Affiliations:** ^1^Functional Genomics Unit, Institut Pasteur de Montevideo, Mataojo 2020, 11400 Montevideo, Uruguay; ^2^Laboratório de Biologia Celular e Ultraestrutura, Instituto de Microbiologia Paulo de Góes, Universidade Federal do Rio de Janeiro, Cidade Universitária, Ilha do Fundão, 21941-590 Rio de Janeiro, RJ, Brazil; ^3^Laboratório de Ultraestrutura Celular Hertha Meyer, Instituto de Biofísica Carlos Chagas Filho, Universidade Federal do Rio de Janeiro, Avenue Carlos Chagas Filho 373, 21941-902 Rio de Janeiro, RJ, Brazil; ^4^Department of Medicine, Faculty of Medicine, Avenida Italia s/n, 11600 Montevideo, Uruguay

## Abstract

At present, noncoding small RNAs are recognized as key players in novel forms of posttranscriptional gene regulation in most eukaryotes. However, canonical small RNA pathways seem to be lost or excessively simplified in some unicellular organisms including *Trypanosoma cruzi* which lack functional RNAi pathways. Recently, we reported the presence of alternate small RNA pathways in *T. cruzi* mainly represented by homogeneous populations of tRNA- and rRNA-derived small RNAs, which are secreted to the extracellular medium included in extracellular vesicles. Extracellular vesicle cargo could be delivered to other parasites and to mammalian susceptible cells promoting metacyclogenesis and conferring susceptibility to infection, respectively. Here we analyzed the changes in gene expression of host HeLa cells induced by extracellular vesicles from *T. cruzi*. As assessed by microarray assays a large set of genes in HeLa cells were differentially expressed upon incorporation of *T. cruzi*-derived extracellular vesicles. The elicited response modified mainly host cell cytoskeleton, extracellular matrix, and immune responses pathways. Some genes were also modified by the most abundant tRNA-derived small RNAs included in extracellular vesicles. These data suggest that microvesicles secreted by *T. cruzi* could be relevant players in early events of the *T. cruzi* host cell interplay.

## 1. Introduction

*Trypanosoma cruzi*, the causative agent of Chagas' disease, is a protozoan parasitewith a complex life cycle, which includes intracellular and extracellular forms that alternate between invertebrate insect vectors belonging to the subfamily Triatominae and mammalian hosts including humans [1, 2]. To cope with these changing environments,* T. cruzi* must undergo rapid and significant changes in gene expression which are achieved essentially at the posttranscriptional level by mechanisms that remain to be completely elucidated [3].

Even though small regulatory RNAs (i.e., microRNAs, siRNAs and piRNAs) have recently emerged as key players in novel forms of posttranscriptional gene regulation in most eukaryotes [4], there is no experimental evidence indicating the presence of canonical machineries associated with small RNA-mediated pathways in* T. cruzi* and other unicellular organisms including* S. cerevisiae*,* L. major*, and* P. falciparum* [5].

In a recent work aimed to identify the presence of alternative small RNA pathways which could contribute to the posttranscriptional control in* T. cruzi*, we reported the presence of homogeneous population of small RNAs derived from mature tRNAs representing from 25 to 30% of the small RNA population [6]. Shortly after, the specific production by* T. cruzi* of different populations of small RNAs derived not only from tRNAs but also from rRNAs and sn/snoRNAs was also reported [7, 8].

More recently, we reported that vesicles carrying small tRNAs and the trypanosomatid's Argonaut protein TcPIWI-tryp [9] were actively secreted to the extracellular medium and acted as vehicles for the transfer of these molecules to other parasites and to mammalian susceptible cells but not to nonsusceptible ones. These data suggested that extracellular vesicles (EVs) shed by* T. cruzi* were not only associated with life cycle transition of epimastigotes toward the infective trypomastigote form, but also associated with infection susceptibility of mammalian cells.

It is now accepted that secreted exosomes and shed microvesicles/ectosomes [10] serve as a means for the delivery of genetic information (e.g., miRNAs and mRNAs) and proteins between cells. Interestingly, these exosomal mRNAs and microRNAs were completely functional in recipient cells, thus playing pivotal roles in cell-to-cell communication [11]. It was therefore possible to speculate that* T. cruzi* extracellular vesicles and their cargo could represent a route of intercellular communication delivering “molecular messages” to others cells aimed to induce coordinated responses to assure parasite survival through both the emergence of infective forms and the establishment of a cellular environment able to facilitate infection. In this respect, it was recently reported that* T. cruzi* trypomastigotes invade 5-fold as much susceptible cells when these are preincubated with purified parasite extracellular vesicles [12]. These results suggest that secreted vesicles from* T. cruzi* and their cargo could act as virulence factors by promoting metacyclogenesis and enhancing host cell susceptibility or both.

In order to gain insight on host-pathogen signaling we analyzed the effects induced by* T. cruzi* shed vesicles and their associated small tRNAs cargo on gene expression of susceptible HeLa cells. By using a microarray approach we report that a large set of genes were differentially expressed upon incorporation of* T. cruzi* shed extracellular vesicles in HeLa cells. The elicited response modified mainly host cell cytoskeleton, extracellular matrix, and immune responses pathways. Furthermore, some of the differently expressed genes were also modified when cells were transformed with specific tsRNAs contained in EVs. Taken together, our data provide significant new insight into the early events of the* T. cruzi*-host cell interplay even before contact between the parasite and host cells is established and in the maintenance of the infection which could conduct us to rethink some concepts in host-pathogen biology.

## 2. Material and Methods

### 2.1. *T. cruzi* Epimastigotes and HeLa Cell Line Culture

*T. cruzi *epimastigotes from the Dm28c clone [13] were maintained in exponential growth phase in axenic culture in Liver Infusion Tryptose (LIT) medium supplemented with 10% fetal bovine serum (FBS) at 28°C, with passages every 4 days. Cells from the HeLa cell line were grown in RPMI medium supplemented with 10% FBS and antibiotics (penicillin 100 U/mL and streptomicyn 100 mg/mL) at 37°C, 5% CO_2_ with passages every 3 days.

### 2.2. *T. cruzi* Microvesicles Purification

Epimastigotes submitted to nutritional stress for 48 h in FBS free RPMI were used as a source of EVs. This nutrient starvation has been recognized as an important condition inducing the emergence of a significant fraction of infective trypomastigote-like parasites [9]. The supernatants of 1 · 10^11^ parasites cultured for 48 hours in FBS free RPMI medium were collected and centrifuged at 2000 g for 15 min to eliminate remnant cells. The 2000 g supernatants were collected and centrifuged at 15,000 g at 4°C for 30 min to remove cell debris and eventual apoptotic blebs. The 15,000 g supernatant was ultracentrifuged at 110,000 g at 4°C for 70 min to pellet small extracellular vesicles. The pellet was washed twice in PBS and further ultracentrifuged at 110,000 g for 1 h. Isolation procedures were evaluated by transmission electron microscopy and quantification of EVs was done by determining the total protein concentration by the Bradford protein quantification assay (Pierce). By these procedures the total protein yield of the small vesicular fraction was about 1.2 *μ*g per  1 · 10^10^  parasites.

### 2.3. Optimization of EVs-Cells Incubation Conditions

For the determination of the incubation time, 0.5 · 10^6^ cells were incubated with 300 ng of the* T. cruzi* EVs preparation for 5 minutes, 30 minutes, 2 h, and 24 h. Following incubation, cells were washed twice with PBS, stained with DAPI, and analyzed by fluorescence microscopy. For the determination of the EVs-per-cell ratio, 0.5 × 10^6^ HeLa cells were grown over cover slips in 6 well plates and incubated with 0, 40, 80, 160, 320, and 600 ng of EVs, for 2 hours at 37°C and 5% CO_2_. Cells were then subjected to FISH assays as previously described [6] for the detection of certain small RNAs known to be present in* T. cruzi*'s EVs. Briefly, cells were washed twice in PBS and then fixed with 4% paraformaldehyde in PBS for 10 min at room temperature, washed twice with PBS, and further incubated in 25 mM NH_4_Cl for 10 min. After washing twice in PBS cells were permeabilized with 0.2% Triton X-100 in PBS for 5 min. Slides were then blocked and prehybridized for 2 h at room temperature in bovine serum albumin 2%, 5X Denhardt, 4X SSC, and 35% formamide (hybridization solution). Hybridization was performed overnight in a humid chamber at 37°C in the presence of 100 nM of the indicated oligonucleotide conjugated to Cy3. After hybridization slides were washed twice in 2X SSC-50% formamide, twice in 2X SSC, once in 1X SSC, incubated with SSC 1X—DAPI (1 mg/mL), and finally washed with 0.5X SSC and 0,1X SSC. For signal amplification, cells were then incubated with an anti-Cy3 antibody (Invitrogen) and revealed with an anti-mouse-TRITC antibody (Invitrogen).

### 2.4. sRNA Transfection of HeLa Cells

HeLa cells were transfected using tsRNA^Leu^ and tsRNA^Thr^ synthetic oligoribonucleotides and their corresponding scrambled sequences as controls. Synthetic oligoribonucleotides were chemically modified to avoid degradation by ribonucleases with terminal phosphorothioate bonds and 2′O-methyl ribonucleotides (IDT Inc.) and were labeled with either Cy3 or FAM. Lipofectamine 2000 (Invitrogen) was used as transfection agent (2 *μ*L/mL in Opti-MEM medium) and probes were used at a final concentration of 20 nM.

### 2.5. Microarray Assays

#### 2.5.1. Sample Collection and RNA Isolation and Processing

For EVs-treated cells, HeLa cells were incubated with* T. cruzi*'s EVs for 2 hours at 37°C and 5% CO_2_. Following this incubation, culture medium containing any remaining EVs was removed, cells were washed with PBS, and fresh culture medium was added. Cells were harvested for RNA extraction at 6, 24, or 72 hours after incubation with EVs. Control cells were incubated with EVs-free medium. Each condition was done in triplicate.

For tsRNA/lipofectamine transfected cells, HeLa cells were transfected with tsRNA^Thr^-FAM or tsRNA^Leu^-Cy3 and their respective scramble controls (tsRNA^Thr^Scr-FAM, or tsRNA^Leu^Scr-Cy3) and harvested 24 hours after transfection for RNA extraction. Control cells were treated only with lipofectamine. Cells transfected with either tsRNA^Thr^-FAM or tsRNA^Leu^-Cy3 were included in triplicate, whereas tsRNA^Thr^Scr-FAM, tsRNA^Leu^Scr-Cy3 and control cells were included in duplicate.

For RNA extraction, cells were harvested, washed twice with PBS, and extracted with TRIzol (Invitrogen) following the manufacturer's protocol and further purified with the Illustra RNAspin Mini Isolation kit (GE Healthcare). Both the integrity and quality of the obtained RNA samples were assayed with the RNA 6000 Nano LabChip in the 2100 Bioanalyzer System (Agilent Technologies). Amplification and labeling of the samples were carried out using the Quick Amp Labeling Kit-one color (Agilent Technologies) following the manufacturer's instructions. All samples were labeled with Cy3. Amplified and labeled RNA samples were purified using the RNAeasy mini kit (QIAGEN) and analyzed on a NanoDrop 1000 Spectrophotometer (Thermo Scientific) for quantification and labeling efficiency.

#### 2.5.2. Hybridization, Washing, Scanning of Microarray Chips, and Data Analysis

Hybridization and washing were done using the Gene Expression Hybridization kit (Agilent Technologies) and the Gene Expression Wash Buffer kit (Agilent Technologies), respectively, following the supplier's instructions. Scanning and primary quality controls were carried out in a High-Resolution C scanner (Agilent Technologies).

The obtained results were analyzed with the GeneSpring Multi-Omics Analysis software, version 12.1 (Agilent Technologies). Fluorescence intensity values were log-transformed and normalized, and differentially expressed genes were determined using an ANOVA test (*P* value ≤ 0.05). Differentially expressed genes were then filtered by fold change (specified in the text) and utilized for pathway analysis using the GeneSpring software or the online available tool DAVID [14, 15].

### 2.6. Real-Time PCR (qPCR)

For validation of the microarray results, 1 *μ*g of RNA was retrotranscribed using Invitrogen's Super Script II Reverse Transcriptase and an oligo-dT primer. GAPDH was used for data normalization. Assays were performed in an Illumina Eco Real-Time PCR System using Roche's FastStart Universal SYBR Green Master. The 2^−ΔΔCt^ method and a *t*-test were used to determine significant differences. The sequences and properties of all the oligonucleotides used throughout this work are summarized in (Supplementary Table 1; see Supplementary Material available online at http://dx.doi.org/10.1155/2014/305239).

## 3. Results and Discussion

### 3.1. Monitoring and Kinetics of Uptake of EVs Cargo by HeLa Cells

**Figure 1:**
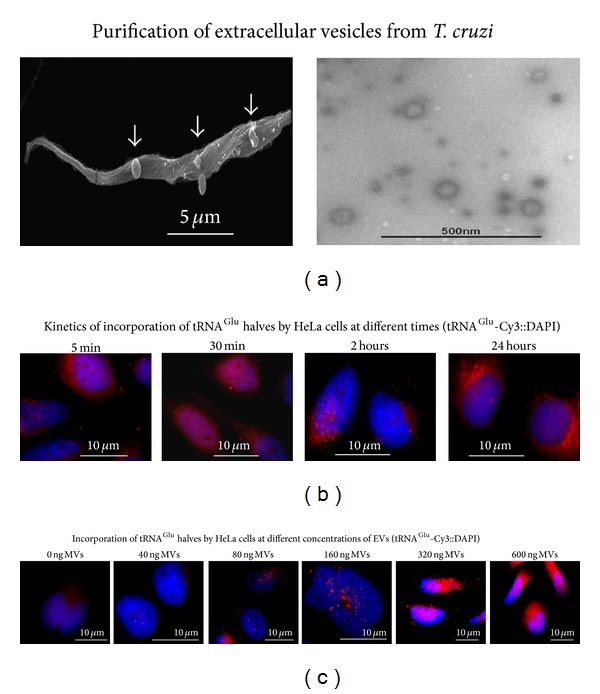
Production of extracellular vesicles by* T. cruzi* and time-course analysis of the uptake of EVs cargo by HeLa cells. (a) Left: scanning electron microscopy of* T. cruzi* epimastigotes showing a parasite with vesicles emerging from the flagellum and the cell body. Right: representative micrograph of purified EVs fraction assessed by transmission electron microscopy. ((b)-(c)) FISH for 5′ halves of tRNA^Glu^ to monitor the incorporation of EVs cargo by HeLa cells exposed to 300 ng of EVs fraction at different times (b) and HeLa cells exposed to different concentrations of EVs ranging from 0 to 600 ng for two hours.

A series of assays was carried out in order to determine the optimum conditions under which the interaction between* T. cruzi* shed EVs and HeLa cells was successful in terms of fusion and EVs cargo delivery. As shown in Figure 1(a), we purified a fraction of EVs shed by epimastigotes submitted to nutritional stress that will be used along the experiments. It is known that epimastigote starvation reproduces the biological environment found in the most posterior portion of the digestive tract of the invertebrate host where metacyclogenesis takes place as an adaptive response to nutritional stress.

For determination of incubation times, HeLa cells at 0.5 · 10^6^/mL^−1^ were incubated in the presence of 300 ng of EVs preparation for different times (Figure 1(b)) and analyzed by FISH for the presence of* T. cruzi* specific intracellular 5′ halves tRNA^Glu^, which was used as a tracer molecule of EVs cargo. Results clearly showed that EVs cargo was incorporated by HeLa cells as early as 30 minutes with a diffuse cytoplasmic pattern which adopted a granular disposition beyond 2 h (Figure 1(b)). According to these results an incubation time of 2 h was then selected as representative for further assays.

To determine the best EVs/host cell ratio to employ in our assays, the same amount of HeLa cells was treated for 2 hr with different amounts of EVs ranging from 0 to 600 ng of the EVs preparation. Cells were then subjected to tRNA^Glu^—derived 5′ halves visualization by FISH. As depicted in Figure 1(c) the treatment with 160 ng was identified as an intermediate condition, where cells did not appear oversaturated. Once we selected an incubation condition of 2 h long with 160 ng of purified* T. cruzi *shed EVs, viability assays excluded deleterious effects on cell performance under culture in these conditions (results not shown). It is important to note that the treatment conditions selected here do not necessarily represent the actual conditions that take place during natural infection and to the best of our knowledge information regarding the amount of EVs produced by parasites during the course of infection remains to be elucidated.

### 3.2. A Microarray-Based Kinetic Analysis Revealed That HeLa Cells Respond to* T. cruzi* EVs with Significant Changes in Gene Expression Patterns Lasting for at Least 72 h

**Figure 2:**
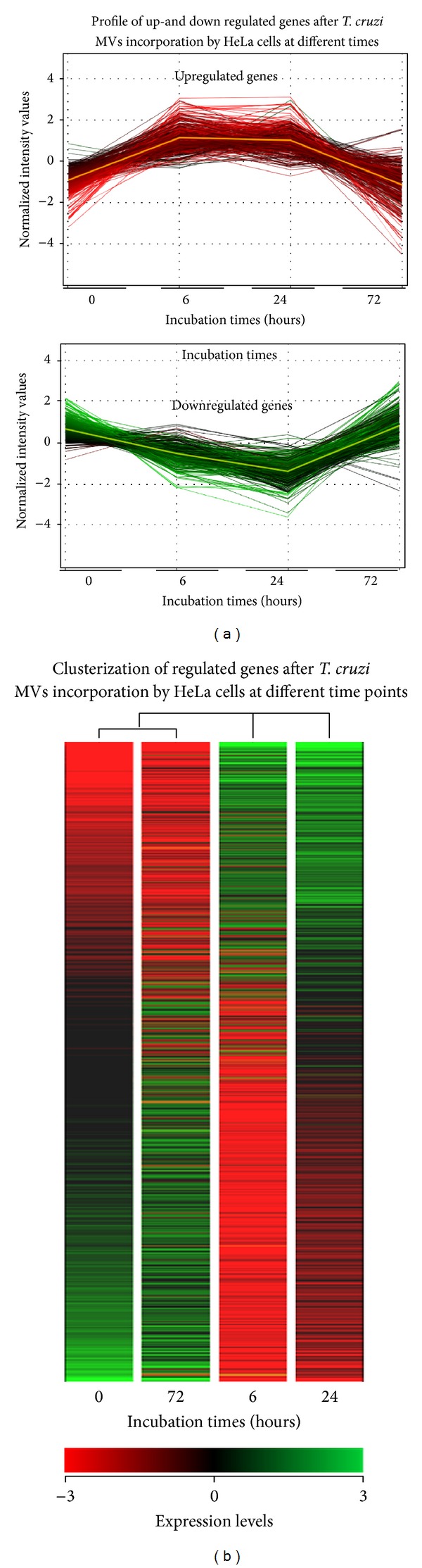
Profile of transcriptomic changes in HeLa cells cocultured with EVs secreted by* T. cruzi*. HeLa cells were exposed to 160 ng of EVs fraction for two hours and analyzed at different time points. (a) Profile of upregulated (upper panel) and downregulated (bottom panel) genes at different times. (b) Cluster analysis of HeLa regulated genes (FC ≥ 3) at different time points (red: underexpressed; green: overexpressed). Experiments were performed by duplicate on three independent samples.

**Figure 3:**
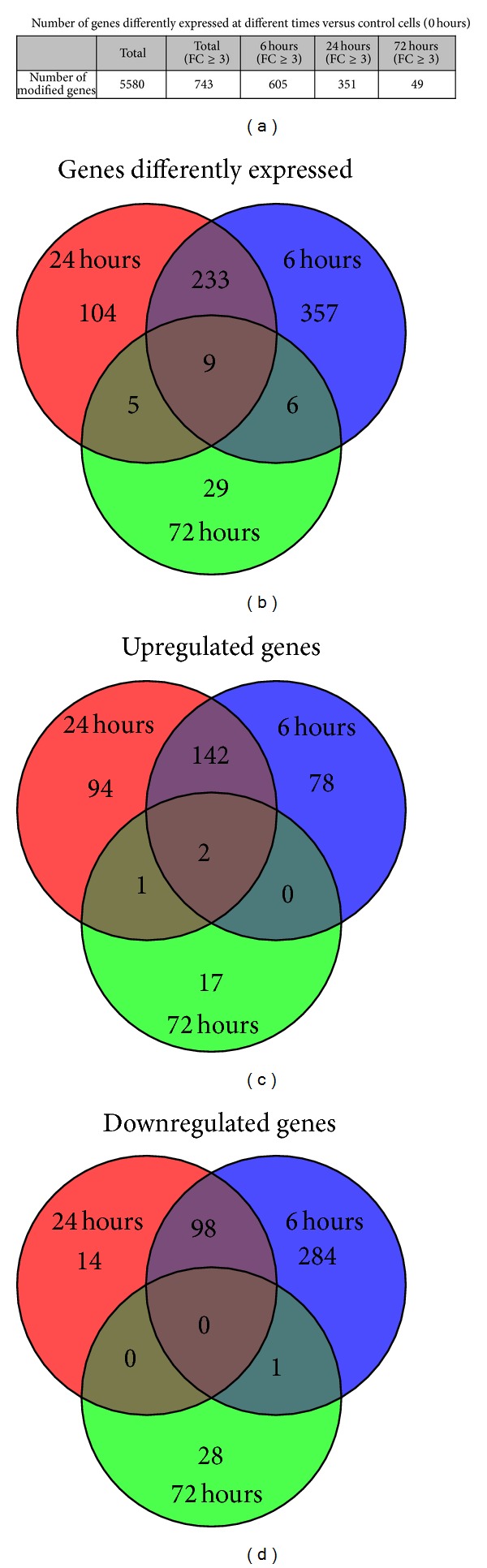
Temporal changes of gene expression in HeLa cells induced by extracellular vesicles secreted by* T. cruzi*. HeLa cells were exposed to 160 ng of EVs fraction for two hours and analyzed at 6, 24, and 72 hours. (a) Table showing the number of differentially expressed genes at different time points. (b) Venn diagrams showing shared and exclusive differentially expressed genes with fold change (FC) ≥ 3. (c) Venn diagrams showing shared and exclusive upregulated genes with FC ≥ 3. (d) Venn diagrams showing shared and exclusive downregulated genes with FC ≥ 3. Experiments were performed by duplicate on three independent samples.

Changes in gene expression profiles of EVs-treated cells were analyzed by triplicate using an Agilent's microarrays platform at three different time points. A total of 743 unique transcripts differentially expressed with a fold change ≥3 and a significance level of ≤0.05 were identified and selected for further analysis (Supplementary Table 2). A graphical view and clusterization analysis (Figure 2) revealed that intact EVs elicit a response in host cells that is more marked at 6 hrs after treatment, is maintained after 24 hrs, but decays towards 72 hrs, where most cellular modified mRNAs returned to basal levels. As depicted in Figure 3(a), the number of total genes differentially expressed at each time point was 605, 351, and 49 at 6 h, 24 h, and 72 h, respectively. A complete picture of the modified genes at different time points is displayed in Figure 3(b) which shows that nearly 40% of the affected genes after 6 hrs were also modified after 24 hrs, whereas only 2.5% remained changed after 72 hrs. The same analysis but now performed separately for up- and downregulated genes revealed a similar behavior (Figures 3(c) and 3(d)). The obtained results seem to indicate that* T. cruzi* shed EVs trigger an important change in the host's cellular gene expression profile.

Recently, Trocoli-Torrecilhas and coworkers described that the treatment of mice with trypomastigote-derived EVs prior to infection caused premature death with an intense inflammatory response while favoring heart parasitism [12]. Later, cells treated with trypomastigote-derived EVs were found to be 5 times more infected by* T. cruzi* than untreated ones [16]. These findings strongly suggest that EVs could play a relevant role during infection. In addition, the uptake of these EVs and their cargo by other parasites as well as mammalian host cells was also described [9]. EVs uptake by other parasites was reported to trigger life cycle transitions and to be involved in promoting susceptibility to infection of host cells [17, 18]. Altogether, evidence gathered so far provide support to the notion that* T. cruzi* secreted EVs in early times may be “preparing” host cells for invasion, through either the interaction with the cell surface or their internalization [16]. Taking into account that the interaction between EVs and host cells would take place previously to direct interaction with parasites themselves, it is possible that the changes that they bring about will take place rather quickly. In addition, if EVs role is that of facilitating parasite entry into the cell, it may not be necessary for the effect of EVs to be sustained over time, since once the infection has been achieved, the parasites themselves represent stimuli enough to continue with the infection process. In the subsequent period of times studied, for example, 24 hours, the changes observed in the host cell could be due to modifications needed to maintain a productive infection once the parasite entered the cell. This could be achieved by modifications in the gene expression of the infected cell itself, as we have seen in the present work, or could be through changes in neighboring cells which enable a proparasitic environment. Of note, in our experimental setting, parasites are absent throughout the entire assay which allows a complete recovery by 72 h.

### 3.3. EVs Secreted by* T. cruzi *Induce a Broad Response Modifying Host Cell Cytoskeleton, Extracellular Matrix, and Immune Responses Pathways

**Figure 4:**
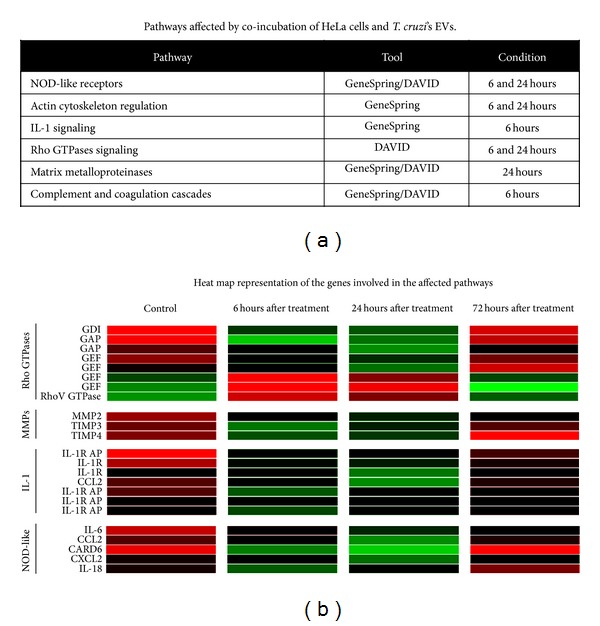
Pathways affected by EVs treatment. HeLa cells were exposed to 160 ng of EVs fraction for two hours and analyzed at 6, 24, and 72 hours. (a) Table showing the cellular pathways affected by EVs treatment. The corresponding time points and the analysis tool used are included. (b) Heat map representation of the modified genes involved in these pathways (indicated at the left of the figure) for each time point (indicated at the top). For the Rho GTPase pathway the role of the modified genes is expressed as GDI (guanine nucleotide dissociation inhibitor), GAP (GTPase-activating protein), or GEF (guanine nucleotide exchange factor). MMP: matrix metalloproteinase; TIMP: tissue inhibitor of metalloproteinases; IL-1R: interleukin 1 receptor; IL-1R AP: interleukin 1 receptor adaptor protein; CCL2: CC-chemokine ligand 2; CXCL2: CXC-chemokine ligand 2; IL-6: interleukin 6; IL-18: interleukin 18; CARD6: caspase recruitment domain family, member 6. Red: underexpressed; green: overexpressed. Experiments were performed by duplicate on three independent samples.

Differentially expressed genes were grouped into functionally related groups of genes using the GeneSpring software and the public bioinformatic resource DAVID. Several pathways were identified as affected by EVs treatment, particularly after 6 or 24 hrs (Figure 4(a)). Some of these pathways were of particular interest in light of the infection-favoring role of EVs derived from stressed epimastigotes [9]. That is the case of the Rho GTPases signaling pathway affected both at 6 and 24 hrs after treatment. Genes belonging to this pathway were modified in a way that would keep this signaling pathway inactive (Figure 4(b)). This family of proteins coordinates and regulates aspects related to cell morphology and motility, through rearrangement of the cytoskeleton. When active, this pathway induces actin polymerization [19]. In fact, the regulation of actin cytoskeleton is one of the pathways identified as affected by EVs treatment at the same time points. Cytoskeleton reorganization has been recognized as one of the main processes that takes place during parasite entry, with actin depolymerization most likely facilitating entry but not retention of parasites within the cell [20, 21]. In this context, EV-triggered depolymerization of cortical actin cytoskeleton in the early stages of interaction would facilitate the initial entry of parasites. Recent work by Mott and coworkers [22] evaluated how host cell mechanics in terms of cytoskeleton remodeling and stiffness were affected by* T. cruzi* trypomastigotes infection and by exposure to shed components present in conditioned medium. Nevertheless, further experiments on EVs-treated cells are necessary to assess whether predicted depolymerization actually takes place at the time points evaluated here.

Matrix metalloproteinases (MMP) are responsible for extracellular matrix (ECM) remodeling. Analysis with both DAVID and GeneSpring revealed that MMPs were altered after 24 h of treatment with EVs. Surprisingly, both MMP and 2 MMP inhibitors (TIMPs) were found to be upregulated (Figure 4(b)). The parasite is known to secrete proteases capable of degrading the ECM, therefore collaborating with the invasion process [23, 24]. In addition, host MMPs have been implicated in the process of tissue damage during infection [25, 26]. One can speculate that EVs stimulate the expression of MMP in order to facilitate ECM degradation before the parasites own proteases, therefore favoring the invasion process. The coexpression of TIMPs may represent the cells response to an unexpected increase in the corresponding MMP activity.

Despite the fact that* T. cruzi*'s EVs seem to aid in the establishment of an infection-favorable cellular environment by eliciting changes in gene expression in response to foreign molecules. In this sense, the NOD-like receptor signaling pathway was found to be activated 6 and 24 hrs after treatment. As depicted in Figure 4(b), mRNAs encoding proteins involved in signal transduction as well as the pathway final products (IL-6, IL-18, CCL2, and CXCL2) were found to be upregulated. It seems likely that cells are able to perceive the presence of strange molecules within the cytoplasm and respond to these signals by producing proinflammatory cytokines and interleukins. Tightly related to the NOD-like signaling pathway is that of IL-1, which was found to be altered 24 h after incubation with EVs. As depicted in Figure 4(b), both the receptor and coreceptor of both types of IL-1 were found to be upregulated as is the proinflammatory cytokine CCL2, a final product of this pathway.

By using a microarray-based approach Manque and collaborators [27] demonstrated that the expression profile of murine cardiomyocytes was greatly affected during the early stages of invasion and infection by* T. cruzi* trypomastigotes. Similar to what we found with EVs incubation, cytoskeleton and ECM remodeling were some of the processes the authors found to be affected, as well as the expression of proinflammatory cytokines and other genes involved in the immune response.

### 3.4. HeLa Cells Respond to* T. cruzi* tsRNAs with Changes in the Expression Levels of Specific Genes

**Figure 5:**
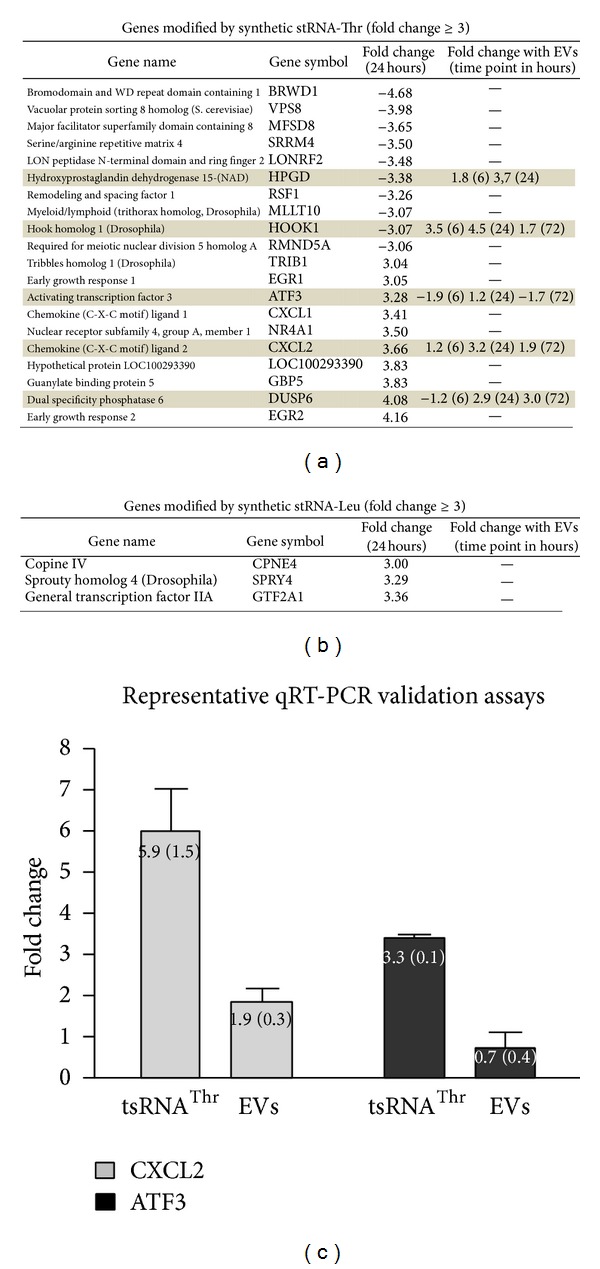
Genes affected by transfection with tsRNA^Thr^ or tsRNA^Leu^. (a) Tables showing the name, gene symbol, and fold change for each of the twenty transcripts whose expression was modified upon transfection with tsRNA^Thr^ and the 3 transcripts modified after transfection with tsRNA^Leu^. (b) Genes which were also modified by EVs are highlighted in grey with the respective fold change.(c) Representative quantitative RT-PCR assays for the CXCL2 and ATF3 genes. Numbers in graphics represent the mean value with their respective SD in brackets. In all cases experiments were performed by duplicate on three independent samples.

Deep sequencing of small RNAs included in EVs from stressed epimastigotes revealed that small RNAs derived from rRNA and tRNA represented about 45% for each. Of note, more than 80% of tsRNAs derived from a restricted group of 4 tRNAs (Leu, Thr, Glu, and Arg) [9]. In order to evaluate their potential effect on host cells, HeLa cells were transfected with Cy3 or FAM-labeled synthetic tsRNA^Thr^, tsRNA^Leu^, and their corresponding scrambled sequences as controls. Transfection efficiency was estimated by fluorescence microscopy, being more than 90% in all experiments. Cell viability was not affected as determined by crystal violet assays (data not shown). A list of genes modified upon transfection with tsRNA^Thr^, tsRNA^Leu^ was obtained by subtracting genes nonspecifically affected by lipofectamine or the corresponding scrambled counterpart. As depicted in Figure 5, transfection of HeLa cells with tsRNA^Thr^ induced significant changes in the level of 20 transcripts at 24 h with a fold change ≥ 3. For tsRNA^Leu^ we only could identify the modulation of 3 transcripts when using a fold change ≥ 3. Further analysis of individual transcripts modified by these tsRNAs revealed that only some of them were also affected by incubation with EVs at any time regardless of their fold change. Indeed, five genes differentially expressed after tsRNA^Thr^ transfection were also affected by EVs treatment (HPGD, HOOK1, ATF3, CXCL2 y DUSP6 in Figure 5). In this respect, the possibility exists that the effect induced by individual components of complex structures as EVs could be masked or modulated by whole changes induced by EVs containing a diversity of different molecules derived from lipids, proteins, and nucleic acids. In agreement with this idea, it can be noted that some of the genes that were upregulated by EVs treatment (e.g., HPGD and HOOK1) were significantly downregulated by treatment with tsRNA^Thr^. Conversely, the ATF3 transcript which was downregulated by EVs underwent an opposite response showing a significant upregulation upon transfection of tsRNA^Thr^. Of note, in the cases of transcripts modified by both EVs and tsRNA^Thr^, the fold change observed was more important for the single small tRNA. It should be also taken into account that the tsRNA concentrations used here do not necessary mimic those found within EVs or in cells after their entry. Finally, the tsRNA entry pathway may not be the same when delivered by EVs or by transfection agents such as lipofectamine. Thus, their intracellular distribution may vary in each case, affecting in turn their access to different cellular compartments, which could explain the observed differences.

Some transcripts specifically modified by both EVs and tsRNA^Thr^ were reported to be relevant in host-parasite biology. The ATF3 gene encodes a cAMP-dependent transcription factor, whose expression is induced by stress signals such as cytokines, lack of nutrients, and bacterial infection. ATF3 is capable of both activating and repressing transcription of target genes involved in cell defense mechanisms and immune response against pathogens [28, 29]. The CXCL2 is a member of the CXC family of chemokines, recognized as key mediators of inflammatory processes [30, 31]. Analysis of these two genes revealed the existence of some evidence of coexpression between ATF3 and CXCL2 which is consistent with their role in immune responses. Finally, DUSP6 is a dual-specificity phosphatase, which belongs to the MAPK phosphatase family, with specificity for extracellular-signal regulated kinases (ERKs), whose activity was associated with adhesion, cell growth, proliferation, cytoskeleton regulation, and survival pathways [32–34].

Transcripts specifically modified by individual tsRNAs derived from threonine and leucine were confirmed by qRT-PCR. A representative experiment for CXCL2 and ATF3 is depicted in Figure 5(c). In all cases, the fold change values obtained by qRT-PCR were significantly higher than values obtained by microarrays quantification.

The verification of CXCL2 and ATF3 overexpression after tsRNA^Thr^ transfection provides evidence that supports the notion that tRNA-derived small RNAs are capable of modulating gene expression. This does not imply that the tsRNA^Thr^ cargo of EVs is solely responsible for the overexpression of these genes, but it does mean that it is capable of doing so. Additional studies should be performed in order to verify if the changes in gene expression observed are due to the direct interaction between tsRNA and mRNA or if it is a “secondary” effect, mediated by other elements present in host cells.

Due to the fact that, like other small RNAs, tsRNAs have been proposed to act as regulators of mRNA levels and stability, we performed a bioinformatic analysis of putative “seeds” or complementary binding sequences for the* T. cruzi*-derived tsRNA^Thr^ in modified transcripts of HeLa cells. This analysis revealed that ATF3, CXCL2, and DUSP6 have putative binding sites for the tsRNA^Thr^  (Supplementary Figure 1). This could imply that these mRNAs have the potential to be direct targets of tsRNA^Thr^. Despite some experimental evidence suggesting [35, 36] that tsRNA was able to regulate gene expression through translation initiation inhibition and possibly direct the specific degradation of mRNAs, this mechanism of action remains to be experimentally validated. It is also possible that the tsRNA^Thr^-mediated upregulation of certain transcripts could be a downstream effect of tsRNAs on other direct targets. Alternatively, binding of tsRNA with its target mRNA could take place and induce upregulation, as it has been reported for certain miRNAs under particular cellular conditions [37]. In this respect, bioinformatic search for possible binding sites on target mRNAs (including 5′ and 3′ UTR sequences) in combination with reporter assays should be conducted to validate any mechanism of action involving direct interaction of tsRNAs with their putative mRNA targets. It is also possible that tsRNAs action could be mediated through RNA binding proteins or other potential molecules.

## 4. Conclusions

In this work we reported that extracellular vesicles secreted by* T. cruzi* epimastigotes undergoing nutritional stress are able to induce epigenetic changes in mammalian cells susceptible to infection. Additionally, several transcripts modified by EVs were also found to change upon transfection of HeLa cells with two of the most abundant tRNA-derived small RNAs included in EVs. These data suggest that* T. cruzi* secreted vesicles could have a high impact in host cells responses against pathogens and that tRNA-derived halves could be one of the molecules inducing these changes. Taken together, these results highlight the relevance of extracellular vesicles and their cargo in early steps of host-pathogen interactions.

## Supplementary Material

Table S1: List of oligoribonucleotides used for transfections, FISH and RT-PCR
Table S2: A complete list of HeLa genes modified by extracellular vesicles at different time points
Supplementary Figure 1: Bioinformatic prediction for target sites for tsRNA Thr in the mRNAs studied showing the binding sites and the corresponding free energy values.
