# Supplementary material for: Gene Expression Changes Induced by Trypanosoma cruzi Shed Microvesicles in Mammalian Host Cells: Relevance of tRNA-Derived Halves
Source: Biomed Res Int. 2014 Apr 9;2014:305239. doi: 10.1155/2014/305239 (PMC4000953; doi:10.1155/2014/305239)
Supplement: Supplementary file 1 — Table S1: List of oligoribonucleotides used for transfections, FISH and RT-PCR Table S2: A complete list of HeLa genes modified by extracellular vesicles at different time points Supplementary Figure 1: Bioinformatic prediction for target sites for tsRNA Thr in the mRNAs studied showing the binding sites and the corresponding free energy values. [file 305239.f1.pdf]

**Oligoribonucleotides for transfections**<br>(***:** Phosphorothioate bonds **–** ***Italics*****:** 2' O-methyl ribonucleotides – **Scr:** scramble oligos)		
**Name**	**Sequence (5’-3’)**	**Fluorophore**
**stRNA-Leu**	A*A*C*_GUCAAGUCUUAGACCACUCGACCAUCUUG_*C*C*	Cy3
**stRNA-Leu-Scr**	A*C*A*_CCAUUACUCGCACUACCAUCCGACGUAGU_*U*G*	Cy3
**stRNA-Thr**	G*A*C*_GGAGGUCGGGGGUUCGAUCCCCCCAGUGG_*C*C*	FAM
**stRNA-Thr-Scr**	G*C*G*_GUGCGCGACGUCCGGUCGGCGAUUCGCAC_*A*G*	FAM
**Oligoprobes used for FISH**		
**tRNA Glu^{UUC}**	CAGGTGTTGTAACCGTTATACCATATC	Cy3
**tRNA Glu^{CUC}**	CGCTTGTAGCCACTATACCAC	Cy3
**tRNA Asp^{GUC}**	CGGGTATACTTACCACTATACTACCGA	Cy3
	**Oligonucleotides used for RT-PCR**	
**ATF3 F**	GGAGCCTGGAGCAAAATGATG	None
**ATF3 R**	GACGATGGCAGAAGCACTCA	None
**LNX2 F**	GCATGAGCCACTCTGCACTA	None
**LNX2 R**	GCCAGGCCAACAAATAACGG	None
**PRAME F**	CAAGCGTTGGAGGTCCTGAG	None
**PRAME R**	TCTGAATGGAACCCCGCAAA	None
**DUSP6 F**	GCGGATCAGCTCTGACTCTT	None
**DUSP6 R**	CCATCCGAGTCTGTTGCACT	None
**CXCL2 F**	ACGGCAGGGAAATGTATGTGT	None

**CXCL2 R**	TCGAAACCTCTCTGCTCTAACA	None
**GAPDH F**	CCAGTGGACTCCACGACGTA	None
**GAPDH R**	GCGAGATCCCTCCAAAATCA	None
