# Supplementary material for: Gene Expression Changes Induced by Trypanosoma cruzi Shed Microvesicles in Mammalian Host Cells: Relevance of tRNA-Derived Halves
Source: Biomed Res Int. 2014 Apr 9;2014:305239. doi: 10.1155/2014/305239 (PMC4000953; doi:10.1155/2014/305239)
Supplement: Supplementary file 3 [file 305239.f3.pdf]

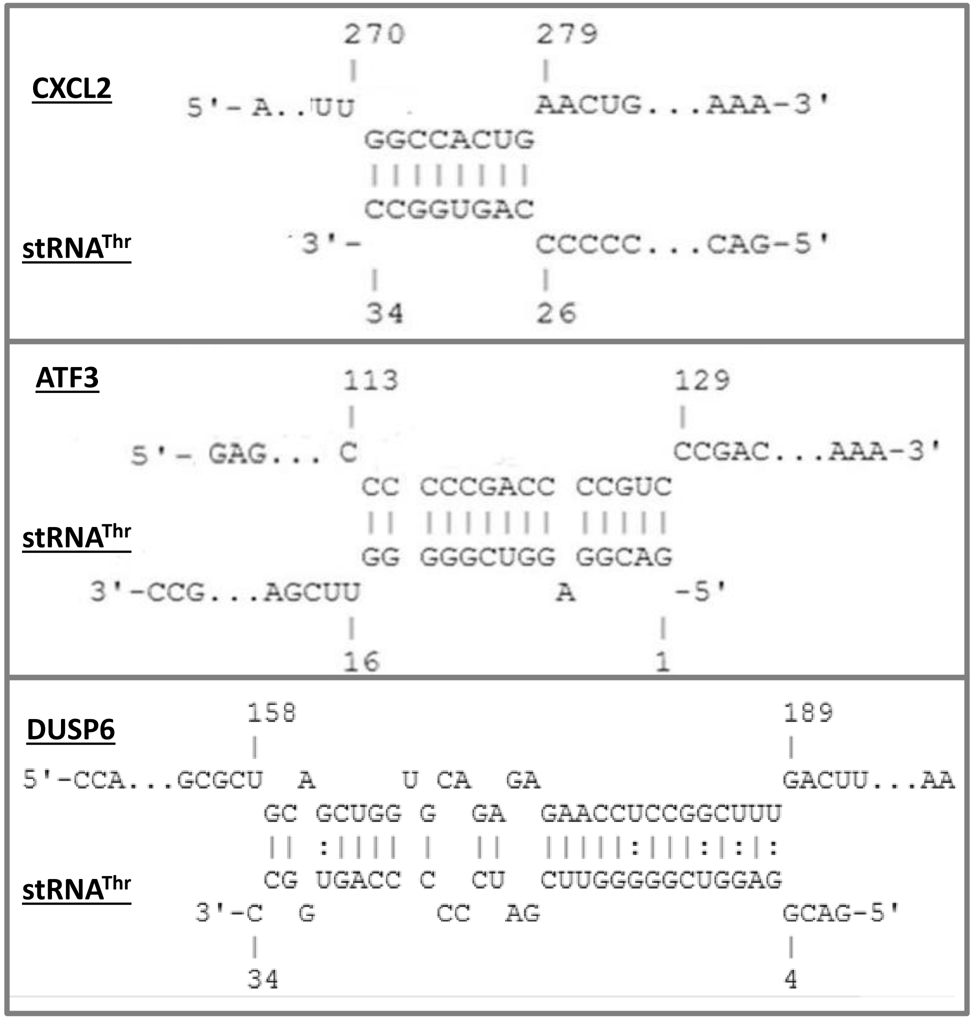

mRNA	Energy (kcal/mol)	Position stRNA	Position mRNA
CXCL2	-11,746	27-34	271-278
ATF3	-11,6	1-15	114-128
DUSP6	-11,807	5-33	159-188

Supplementary Figure 1: Bioinformatic prediction for target sites for tsRNA Thr in the mRNAs studied. Bionformatic prediction (http://rna.informatik.uni-freiburg.de:8080/v1/IntaRNA.jsp, IntaRNAv1.2.5) of putative binding sites for $tsRNA^{Thr}$ in ATF3, CXCL2 and DUSP6 transcripts which were found modified after its transfection to HeLa cells. The Figure shows the binding sites and the Table the characteristics of the binding. The corresponding free energy values and the respective positions of interacting sequences are showed in the attached table.
